# The effects of language preference and home resources on foundational literacy retention during school holiday closures in Ghana: Lessons from the Complementary Basic Education Programme

**DOI:** 10.1007/s11125-021-09590-6

**Published:** 2021-12-03

**Authors:** Kwame Akyeampong, Emma Carter, Pauline Rose, Jennifer Ryan, Ricardo Sabates, Jonathan M. B. Stern

**Affiliations:** 1 School of Education, Childhood, Youth and Sports, The Open University, Stuart Hall Building, Walton Hall, Milton Keynes, MK7 6AA UK; 2 Faculty of Education, University of Cambridge, 184 Hills Road, Cambridge, UK; 3 RTI International, Research Triangle Park, NC USA

**Keywords:** Covid-19 school closure, Literacy, Mother-tongue education, Learning, Equity, Ghana

## Abstract

This article assesses the extent to which children’s language preference and their home environment matter for literacy retention. Using data from the Complementary Basic Education (CBE) program in Ghana, the authors found that large numbers of disadvantaged students reverted to not even being able to read a single word following school closures over a four-month holiday period. Widening literacy gaps were found for girls who reported they did not receive instruction in a language that they understood or did not have the resources, support, or activities at home to enable them to continue to learn while schools were closed. For boys, widening literacy gaps were only influenced by resources, support, or activities at home, but not by language preferences. The article findings suggest that schools and teachers must pay closer attention to language preference, particularly for girls, in order to ensure that language of instruction is not a barrier to literacy retention. The article also provides further evidence to support the growing claims that home supports are essential for reducing inequities in learning outcomes during school closures.

Questions about how to ensure continuity of learning during school closures have come to the fore in the context of the Covid-19 pandemic. This is particularly the case in low and lower-middle-income countries (LMICs), where many children already lack basic foundational skills. While it is still too early to fully assess the impact of school closures during the pandemic, evidence from prior school closures can be informative for the current context. In this article we focus on the effects of closures during the holiday break between school years in Ghana, with a particular focus on children’s language preference and home environment.

For early years education, the use of resources written in the child’s own language enables them to understand the basic properties of literacy acquisition and ultimately smooth the transition to other languages of instruction (Carter et al., 2020a, b; Cummins, 1979). Many early learning programs in multilingual environments use local languages as a means to improve foundational reading skills (Brock-Utne, 2010; Piper et al., 2016; Trudell, 2009), not just through pedagogical approaches but also through the content of the curriculum (Brock-Utne and Alidou, 2011). Children are found to become more actively engaged in education when they are taught in a language they understand (Brock-Utne and Alidou, 2011). Yet, many children in multilingual environments find it difficult to understand their lessons and to grasp the instructional content provided by teachers. While education is usually given in another language due to policy reasons or the preferences of parents (Trudell, 2009), consideration must be given to the language preferences of children as well. This is likely to be particularly important when assessing the effects of school closures, which can influence literacy retention over time.

The CBE program in Ghana presents an interesting case for exploring the implications of language preference and home environment for literacy acquisition and retention. CBE is designed to cater to children ages 8–14 who have either not had the opportunity to attend formal primary school or have dropped out early due to personal disadvantages they face. CBE provides them with basic literacy and numeracy instruction in one of eleven mother-tongue languages. The 9-month accelerated learning program is aimed at delivering the knowledge and skills required for children to successfully transition to nearby government primary schools upon completion.

The language policy in Ghanaian primary schools stipulates that teaching in the first three years of primary education should be in the child’s own language. In the fourth year of primary school, the language of instruction shifts to English, and the local language is taught as a subject. Even where instruction is in local languages, in multilingual environments, children may not be learning in their own language. The CBE program is designed to offer children instruction in a local language, but this may not always be the most familiar language (or language of preference) for the child. This has potential implications for their learning, which may be exacerbated when schools are closed and available learning resources are in a language different from their own.

Our analysis of the transition between the CBE program and formal primary schools provides insights into the potential linguistic challenges children face during school closures. After spending 9 months learning in a local language in the CBE program, children make the transition to local government schools. During this transition, they spend about 4 months out of school, and foundational literacy loss may be expected. We expect children who have been learning in their preferred local language to retain more foundational literacy during this transition time than children who were taught in other local languages. Literacy retention during this time out of school might also be affected by access to learning materials and support at home. In addition, the extent to which girls and boys are engaged in household chores as well as experiencing gendered cultural practices may also differentially impact their foundational literacy retention. We next explore these three issues empirically.

Foundational literacy loss during grade transition periods in early primary school has been well established in many high-income contexts. In the United States, for instance, primary-school-aged children, particularly those from low-income backgrounds. have been found to suffer formal or academic learning loss as a result of time out of school during the transition (Fairchild, 2002; Kuhfeld, 2019). The Education Endowment Foundation (2020), which gathered evidence from 11 studies from the Global North to estimate the academic learning loss as a result of time out of school during long school holidays, found that children from disadvantaged backgrounds are likely to be around 36% worse off than their more advantaged peers as a result of this time out of school.

Evidence of foundational literacy and numeracy loss resulting from the transition period between grades is also emerging in studies from the Global South. A study by Slade et al. (2017) for Malawi showed that long breaks between academic years had the same negative effects on foundational literacy loss. Children’s literacy and numeracy losses were similar in magnitude when they transitioned from primary school grade 1 to grade 2 and from grade 2 to grade 3. However, there were no gender differences in such losses. Sabates et al. (2021) found that about 66% of previous numeracy gains during the CBE program were lost during the four-month transition period to government public schools. Carter et al. (2020a) study further revealed that low-achieving boys and girls were affected by foundational learning loss in numeracy, amounting to 60% and 64% of previous gains, respectively, during the transition from CBE to government school. During school closures due to Covid-19, Kaffenberger (2020) estimates that about one third of learning is expected to be lost for children in grade 3. In addition, the expected foundational learning loss is likely to accumulate over time if there are no mitigating interventions. None of these studies have explored the role of language preference of children for learning in mitigating learning loss while they are out of school.

## Objective and research questions

Our article contributes to the literature reviewed above on learning loss during grade transitions by examining more specifically whether foundational literacy loss following school closures depends on children’s preferences for mother-tongue language of instruction, as well as the availability of learning resources and support at home, given that these may be particularly relevant for maintaining literacy acquisition.

For this analysis, we used the four-month transition period between end of the CBE program and the start of formal education in government schools to estimate the extent to which foundational literacy retention is greater for children who have preference for instruction in their own language. We also estimate the extent to which foundational literacy retention depends on resources and support for learning at home. Empirically, we used longitudinal data from the CBE program to identify learning gains in letter-sound identification and reading comprehension over the nine-month period of the CBE program (with endline scores in June 2017), and measured these foundational literacy skills again at the start of entry into government school (October 2017). The research questions of this article are:

1. What was the loss in foundational literacy experienced by children who participated in the CBE program over the four months’ transition period prior to entry into government schools?
2. What is the relationship between the child’s language preference in learning and continuity of foundational literacy during the transition?
3. What role did home learning support and resources play in mitigating loss in foundational literacy during this transition period?

The above questions are explored by gender to investigate potential differences in learning loss experienced by boys and girls.

## Methodology: Description of the sample

This study is based on data collected during a longitudinal study of the CBE program in Ghana conducted from 2016 to 2018, funded by the United Kingdom Foreign Commonwealth and Development Office. We collected data from a stratified random sample from 40,000 students enrolled in the CBE program in September 2016. Stratification was done by language of instruction, which was determined by region and the provision of the CBE program by implementing partners. The original sample consisted of 2,360 children located in the Northern region (66%), Upper West (12%), Upper East (11%), Brong Ahafo (9%), and Ashanti (2%). Throughout the study, four rounds of data collection were completed: at the beginning of the CBE program in October 2016, the end of the CBE program in June 2017, the beginning of government school in October 2017, and the end of the first year in government school in June 2018. Over this time, sample attrition was high due to some children not continuing to formal schools following the CBE program, dropout from formal school, and migration and absence at the time of data collection (irregular attendance is high due to seasonality and household chores). Carter et al. (2020b) demonstrated that students with data available across all four time periods were more likely to be higher achievers, miss fewer school days, and be more engaged with their learning activities than students who dropped out of the program.

For the purpose of estimating loss in foundational literacy, we restricted our sample to students who were tested in the same language in which they studied at the end of the CBE and at the start of the first year in government schools. Nearly 47% of the CBE students changed their language of instruction when they transitioned into government schools. Since these students were tested in one local language at the end of the CBE program and another at the start of formal mainstream school, any literacy losses during the transition period are likely to be confounded by changes in linguistic familiarity between the two languages (Carter et al., 2020b). Therefore, we restricted the sample to those students for whom the official language of instruction as reported by the CBE program was the same language as that used for teaching in the early grades of primary school. This corresponds to 665 children, as indicated in Table 2.

The fact that the official language of instruction in government schools is the same as the one used by instructors of the CBE program is not a guarantee that children speak that language at home. As indicated in Table 1, only 40.6% of children reported that the language the instructor during the CBE program was the same as their own language; 43% indicated that they were able to understand the language used by the CBE instructor, whereas 74.5% reported a preference for mother-tongue education. This highlights the fact that, although the CBE program supports the use of local language, in practice this may not always be possible due to multiple languages being used within a community.

**Table 1:** Descriptive statistics of main variables: sample with complete information and sample who transitioned into same language

Variables		Description	Sample complete trajectories	Sample same language	Sig
Language	Mother Tongue (MT)	% prefer to learn in MT	79.3	74.5	**
	Teacher	% language use by CBE teacher easy to understand	45.9	43.3	
		% language use by CBE teacher same as child's language	44.6	40.6	**
Home learning support	Time study	% have time to study at home	68.7	71.2	*
	Asking for support	% asked most time / always for help to adults at home	21.5	21.7	
Home learning resources	Activities at home	% with reading of counting activities at home	73.1	70.1	*
	Reading Materials	% with books or reading materials at home	72.6	68.2	**
	TV	% with TV	15.6	18.4	**
	Radio	% with radio	52.2	50.7	
	Mobile Phone	% with mobile phone	72.5	66.6	**
Controls	Gender	% female	49.2	46.0	
	Lessons easy	% found most of the lessons easy during the CBE	35.8	34.0	
	Effort	% most of the times tried hard during CBE	53.4	46.5	**
	Work	% working outside of the home (paid or unpaid)	43.5	35.6	**
	Age	Average Age (sd)	10.3 (2.2)	10.8 (1.9)	
	HH size	Average household size (sd)	9.9 (5.7)	8.3 (4.3)	*
	Attendance	Average missed days at school (out of 5) and (sd)	1.1 (1.2)	0.9 (1.6)	*
	Grade placement	% placed at grade 4 and above	54.6	64.9	**
	Electricity	% access to electricity at home	33.7	38.8	**
	Poverty	% with less money than others in village	63.6	55.6	**
	Sample size	Number of observations	1166	665	

*Note:* Asterisks *, ** indicate statistical significance at 5% and 1% level, respectively. The sample with complete trajectories consists of those for whom there is full information across 4 time periods. whereas the sample with the same language is a subsample of those who transitioned into the same language from CBE to government schools. The proportion of female students in the same language sample is 46%.

*Source:* CBE Monitoring and Evaluation 2016–2018.

Table 1 shows some differences between children about whose learning trajectories we have full information (used by Carter et al., 2020a, b) and those in the subsample used for this study. In particular, compared with the full sample, children in our restricted sample were less likely to work outside of the home, missed fewer days of school, and were placed in higher grades relative to children whose language of instruction changed between CBE and government schools. While our restricted sample consists of children who were more likely to have a television at home and to be living in households with access to electricity, these children were less likely to have access at home to reading, writing, and counting activities or books. Therefore, there is heterogeneity in the sample, which must be considered when interpreting the results.

## Zero scores in literacy

The main outcome of interest for our study is student performance in foundational literacy. We obtained results using an Early Grade Reading Assessment (EGRA) that was adapted for 11 local Ghanaian languages for this activity (To date, EGRAs have been administered in more than 120 languages across at least 75 countries. See Carter et al. (2020b) for more information about the test used). We focus on two measures that were selected from the range of EGRA literacy subtasks administered during the CBE program (rounds 1 and 2 of data collection) and in formal school (rounds 3 and 4 of data collection). These measures are letter-sound identification and reading comprehension. Given the slight adjustments in these EGRA tests over the testing periods, we have more confidence that these measures are able to capture changes over time, particularly when using “zero scores” as an indicator of non-performance in these subtasks.

By focusing on changes in the proportion of children who are unable to correctly identify a single letter sound or answer a single comprehension question, we are able to provide important insights into the impact of literacy loss for children struggling with tasks at either end of the difficulty spectrum (i.e., letter sounds is an introductory reading task while reading comprehension is the ultimate goal of early grade literacy). Children who are unable to correctly identify any items from these tasks are arguably at the greatest risk for falling behind their peers, and it is therefore important to highlight the factors that contribute to this.

## Key factors related to foundational literacy loss

We estimated the potential role of three factors as enablers of continuity in learning between home and school during the transition period. These factors are preference for mother-tongue education, availability of home learning support, and home learning resources.

Three indicators relating to children’s preferences for language learning were recorded on four-point scales (never, sometimes, most of the time, always), but for empirical analyses, we reclassified them into two categories: never/sometimes and most of the time / always). All questions refer to learning during the CBE program. The first indicator relates to children’s ease of learning through their own language, which is captured in the following statement: “*I found learning easier when I was taught MOSTLY in my mother tongue*”. The second indicator relates to the language used by the teacher and whether this made it easier for the children to understand the lesson. This is captured in the following statement: “*The language the teacher used was easy for me to understand*”. The last indicator relates directly to children’s responses when the language used in class was their own language: “*The language the teacher used was my own language*”. All questions were read to the children by trained enumerators using local languages.

Availability of home learning support was also obtained from children’s self-reported answers to the following statements read by enumerators: “*When I did not understand things at school I asked my mother or a female adult*”, and “*When I did not understand things at school I asked my father or a male adult*”. As with the questions associated with language preference, we created a dichotomized variable for our analyses (i.e., those who never or sometimes ask an adult for help versus those who ask most of the time or always). The other indicator relates to whether the child was given enough time to study at home. This came from the statement “*I was not given enough time to study and review at home*”, which we reclassify into a binary yes/no to indicate whether enough time was given to study at home.

Availability of home learning resources was obtained from indicators that included whether children had access to activities involving reading, writing, or counting as well as the availability of books or other reading materials. We also determined whether there was a television, radio, or mobile phone at home.

In order to identify if there were any gender differences in the effects on literacy retention of language preferences and home learning resources, we also performed our analyses separately for boys (54% of the sample) and girls (46% of the sample).

## Control variables

In addition to the main factors that are the focus of this article, the longitudinal study of the CBE children contains several important indicators related to foundational literacy losses and are therefore used here as control variables. These are the children’s ages (from 8 to 15 years) and the grade in which children were placed in government schools after the transition period (between primary 2 and primary 6). We also included self-rated opinions of student effort obtained from the statement “*I tried hard to learn my lessons*” and the difficulty of the lessons obtained from the statement “*I found most lessons easy when I was at school*”. These statements were read to the children by enumerators using local language. These factors help to account for perceptions about learning that are associated with both learning in a different language and potential foundational literacy loss during the transition.

We included an indicator for school attendance measured by the number of days the child said that they attended school in the week prior to the survey—a common approach for measuring attendance in demographic and health household surveys. In order to account for the potential role of sociodemographic factors and resources available in the household that may mitigate or intensify foundational literacy loss, we included household size, whether the household had access to electricity, whether the child reported doing any work outside the home (paid or unpaid), and whether the child ranked their household among the poorest in the community (relative to average) or among the richest). All household level information was reported by children. Items were designed and piloted to ensure that questions could be answered by children when read by enumerators. The questionnaire was administered to the children individually and orally in their local language.

## Analytical approach

In order to estimate the relative loss in foundational literacy during the transition period we used ordinary least-squares regression. Specifically, we estimated the conditional change in foundational literacy captured by the parameter β_1_ in the following equation:

1 $$ L_{it} = \beta_{0} + \beta_{1} Time + \beta_{2} F_{i} + \gamma X_{it} + e_{it} $$

where *L* is the proportion of zero scores in letter-sound identification or in reading comprehension child *i* in time *t*; Time is a measure before and after the transition—in other words, at the end of one academic year and the start of the next. *F* and X stand for the factors and control variables that we are using to estimate the conditional model.

In order to estimate preference for learning in their own language, as well as factors related to home learning support and resources, we added to equation (1) an interaction term between Time and Factors, which then captures the relative difference in foundational literacy loss between different groups. This is demonstrated by the following extension to equation (1):

2 $$ L_{it} = \beta_{0} + \beta_{1} Time + \beta_{2} F_{i} + \beta_{3} F_{i} |Time + \gamma X_{it} + e_{it} $$

where the parameter β_3_ is equivalent to the difference-in-difference (DID) estimator. In equation (2), β_1_ continues to measure the conditional average foundational literacy loss during the transition but this time for children with a specific combination of factors. β_2_ measures the average difference in zero scores at the end of the CBE program between different groups of children according to the factors of interest. In other words, β_2_ measures how different these children were in their foundational literacy before the transition period (and hence time at home) started. As noted, these models were estimated for the restricted sample of children for whom the language of instruction as reported by the CBE program was the same as the official language of instruction in the government school. All models were also estimated by gender.

## Results

What is the loss in foundational literacy experienced by children who participated in the CBE program during their transition to government schools?

We start by providing an overview of the overall trajectory in zero scores in literacy subtasks to contextualize the learning loss during the transition. For simplicity, we refer to students who are unable to identify any items from a given task (i.e., those with zero scores), as *nonperformers*. Therefore, throughout these results, it is important to keep in mind that lower percentages are preferable (as the goal is to decrease the proportion of nonperformers in letter sounds and reading comprehension). At the start of the CBE program, 11% of children in the estimation sample were unable to identify any letters, and 61% were unable to answer a single reading comprehension question. By the end of the CBE program, the proportion of nonperformers was reduced to 4.5% for letters and to 29% for comprehension. This constitutes an improvement of more than 50% for both subtasks. However, during the four-month period when children were not in school, that is, between completing the CBE program and starting government school, many of the gains had been eroded. The proportion of nonperformers in letters increased to 9%, and those who were unable to comprehend what they read increased to 44%.

**Figure 1:**
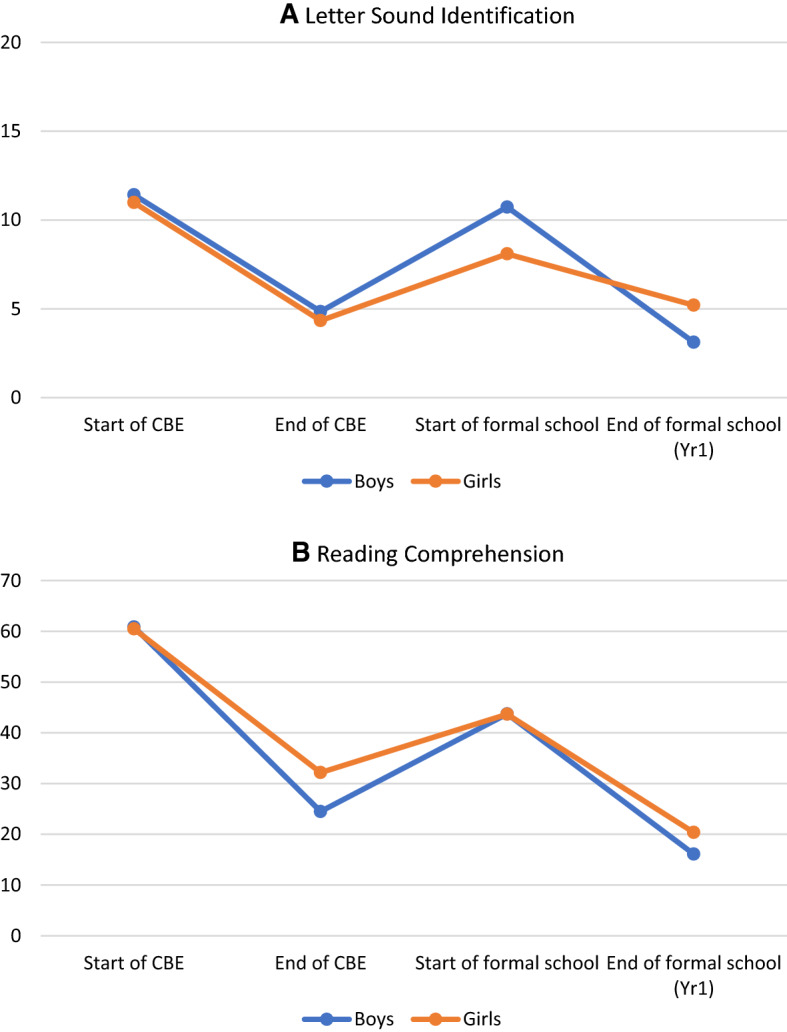
Proportion of zero scores in literacy subtasks over time. *Source:* CBE Monitoring and Evaluation 2016–2018.

While reductions in letter-sound nonperformers followed the same trajectory for boys and girls during the CBE program (Figure 1, Panel A), the loss during transition was slightly worse for boys. An estimate of the unconditional literacy loss in zero scores for letter-sound identification for boys is 7.2% (standard error 2.2%; p-value < 0.01) and for girls is 5.1% (standard error 1.8%; p-value < 0.01). In terms of reading comprehension, the unconditional literacy loss for boys is 20.7% (standard error 2.5%; p-value < 0.01) whereas for girls it is only 12.9% (standard error 3.6%; p-value < 0.01).

In order to assess the magnitude of these literacy losses for boys and girls, we compare them to what they learned in each of these subtasks during the CBE program (i.e., during the 9 months in which they were enrolled in the CBE program). For instance, both boys and girls in the CBE program made improvements in letter-sound identification by reducing their zero scores by an average of about 8%. However, during the transition period, boys lost about 89% of this improvement while girls lost about 56%, which means boys' foundational literacy loss was worse than girls'. Similarly, during the CBE program, boys improved their reading comprehension by lowering their zero scores by 39%. Girls also saw an improvement of 32%. During the transition period, however, boys lost about 52% of the gains they had made, whereas girls lost only about 42% of their gains. In other words, boys seem to lose more of their gains in letter sounds and reading comprehension during transition than girls. A different study by Carter et al. (2020a) found that low-achieving girls in the CBE programme are at a significant disadvantage relative to low-achieving boys. Differences between our analysis here and that of Carter et al. (2020a) may be due to the sample restriction and the fact that we do not focus exclusively on low-achieving children.

There are two important findings to highlight. First, literacy loss during school closure is higher for more basic literacy skills, in this case, letter-sound identification. Second, compared to boys, girls retain more literacy during time out of school in both letter-sound identification and reading comprehension.

## What is the relationship between a child’s language preference and continuity of foundational literacy during transition?

In this section, we build on the overall estimates of foundational literacy loss during school closure in order to determine the extent to which language-related factors are associated with relative losses in zero scores. More specifically, we have included three factors as predictors of loss in our DID model: student’s preference for learning in a mother tongue, whether or not the teacher’s language was easy to understand, and whether or not the teacher used the same language as the child. The results for two models (using zero scores in letter identification and reading comprehension as dependent variables), estimated for all children as well as by gender, are displayed in Table 2. The first result to highlight is the conditional average literacy loss for children who did not prefer to learn in their own language, who did not find the language used by the teacher easy to understand, and who reported that the language used by the teacher was not the same as theirs. These children have an estimated 6.4% increase in zero scores for letter-sound identification and 35.6% for reading comprehension. Here we notice significant differences by gender, with boys appearing more disadvantaged in terms of the simpler task of non-performance in letter sounds while girls are more disadvantaged in the higher-skilled task of reading comprehension. Specifically, for boys, there is an estimated 10.1% increase in the proportion of nonperformers in letter sounds, whereas for girls it was 3.7% (and not statistically significant). For reading comprehension, the average literacy loss for boys was 30.2%, whereas for girls it was 39.1%. It is important to recall that these are boys and girls who reported challenges with their mother tongue and not those at the average level reported in the previous section. It is also important to highlight that these are conditional averages, whereas in the previous section we presented unconditional trajectories.

**Table 2:** Learning loss during transition time: Relative zero scores in literacy by language (by gender)

Variables	All		Boys		Girls	
	Letter identification	Reading comprehension	Letter identification	Reading comprehension	Letter identification	Reading comprehension
	[1]	[2]	[3]	[4]	[5]	[6]
Average learning loss (not preferred language)	6.419*	35.621*	10.103*	30.233**	3.767	39.117**
	**(2.866)**	**(5.192)**	(4.066)	(8.216)	(3.902)	(6.779)
Prefer to learn in MT	0.192	6.697	0.443	− 0.489	0.149	11.380*
	(1.954)	(4.085)	(2.725)	(6.173)	(2.849)	(5.605)
**DID: prefer MT relative to no MT**	2.554	− 15.754**	− 2.987	− 11.013	7.077	− 19.183*
	(3.397)	**(5.956)**	(5.136)	(8.985)	(4.409)	**(8.043)**
Language use by teacher easy to understand	0.316	4.917	1.043	2.628	− 0.484	7.529
	(2.179)	(4.367)	(3.581)	(6.292)	(2.502)	(6.045)
**DID: Language used by teacher easy relative to not**	− 0.604	− 15.147*	1.945	− 3.376	− 3.075	− 25.066**
	(3.361)	**(5.973)**	(5.327)	(9.005)	(4.143)	**(7.992)**
Language use by teacher same as child	2.567	10.804*	1.722	6.524	4.514	13.694*
	(2.281)	(4.393)	(3.446)	(6.343)	(3.014)	(6.065)
**DID: Same language used by teacher relative to not**	− 7.014*	− 7.095	− 8.024	− 5.717	− 6.263	− 8.844
	**(3.301)**	(6.015)	(4.980)	(8.941)	(4.364)	(8.153)
Controls home support and resources	Yes	Yes	Yes	Yes	Yes	Yes
Other controls	Yes	Yes	Yes	Yes	Yes	Yes
Constant	20.073**	59.953**	26.692*	65.841**	12.811	61.659**
	(7.263)	(13.706)	(10.542)	(19.710)	(10.324)	(18.748)
Observations	665	665	359	359	306	306
R-squared	0.091	0.187	0.115	0.214	0.104	0.193

*Note:* Robust standard errors in parentheses (DID parameters) may be interpreted as the *relative loss* within factors. Controls for home learning support and resources and other controls included in the model (results not shown here). Asterisks *, ** indicate statistical significance at 5% and 1% level.

*Source:* CBE Monitoring and Evaluation 2016–2018.

***p < 0.01, **p < 0.05, *p < 0.1

Regarding our DID estimates (bolded variables in the table), we find that several language factors are significantly associated with changes in relative literacy loss. Overall, the associations tended to be larger for the more difficult task of reading comprehension than they were for letter identification. First, children who prefer to learn in their own language had a smaller literacy loss in reading comprehension (16% lower zero scores) relative to those who do not. Second, children who reported that the language used by the teacher was easy to understand fared better than those who reported difficulties understanding the language of their teacher; their zero scores in reading comprehension were about 15% lower. With regard to letter identification, children who reported that the language used by the teacher was the same as their own language had a lower literacy loss (the proportion of zero scores was about 7% lower) during their time at home relative to those who reported their teacher used a language different from their own. However, we notice differences in the significance of these results for boys and girls. In reading comprehension, girls who prefer to learn their own language were able to lower their zero scores by about 19%, and those who found the language of the teacher easier (i.e., similar to their own language) were able to lower their zero scores by about 25%. Interestingly, for boys, we did not find significant differences in reading comprehension or letter identification zero scores as a result of their reports on language factors. This may be an indication of the lesser attention girls, particularly low-performing girls, receive in class, as qualitative evidence from classroom observations suggests (Akyeampong et al., 2018).

## What role do home learning support and resources play in mitigating loss in foundational literacy during this transition period?

In order to respond to the question of the role of home learning support and resources in mitigating literacy loss, we estimated a model including support for learning at home as well as availability of learning resources at home for five separate indicators, as shown in Table 3. Children who reported not having learning support or activities at home were more likely to be nonperformers. The conditional average literacy loss for children who reported no learning support or activities at home was a 12.6% increase in zero scores for letter identification. There are again significant gender differences. Lack of learning support or activities at home is more likely to affect boys who are nonperformers compared with girls. Boys have a conditional average literacy loss in letter-sound identification of 19.7% (relative to other boys) and girls only 6.3% (relative to other girls). The effects on the more difficult task of reading comprehension are higher overall (a 26.5% increase in zero scores). In this case, the gender pattern is reversed. The conditional average literacy loss for boys without any home support or learning activities at home is 20.8% (relative to other boys) and for girls 29.4% (relative to other girls).

**Table 3:** Learning loss during transition time: relative zero scores in literacy by home learning support and resources (by gender)

Variables	All		Boys		Girls	
	Letter identification	Reading comprehension	Letter identification	Reading comprehension	Letter identification	Reading comprehension
	[1]	[2]	[3]	[4]	[5]	[6]
Average learning loss (no home learning supports or	12.612**	26.458**	19.740**	20.805	6.292	29.408**
resources)	(4.601)	(7.464)	(6.954)	(11.507)	(6.034)	(10.157)
Time to study at home	1.239	− 8.945*	5.620**	− 12.868	− 1.192	− 6.889
	(1.843)	(4.354)	(2.096)	(6.680)	(2.670)	(5.955)
**DID: time to study relative to no time**	0.132	9.692	− 3.257	13.015	3.140	7.043
	(3.182)	(6.060)	(4.179)	(9.232)	(4.625)	(8.246)
Ask for help most of the times	− 1.324	2.552	0.336	3.224	− 2.777	1.187
	(2.123)	(4.644)	(4.181)	(6.604)	(2.174)	(6.783)
**DID: most times ask relative to sometimes/never ask**	− 0.886	− 26.128**	− 0.532	− 23.085**	− 1.813	− 27.332**
	(3.369)	**(5.934)**	(5.550)	**(8.461)**	(4.172)	**(8.488)**
Literacy/numeracy activities	4.017	12.992*	5.753*	18.218*	3.093	10.044
	(2.079)	(5.771)	(3.239)	(7.141)	(2.742)	(8.618)
**DID: Learning activities relative to none**	− 4.754	− 17.718*	− 15.489*	− 22.516*	4.789	− 13.519
	(3.929)	**(7.702)**	**(6.061)**	**(10.596)**	(4.843)	(10.881)
Reading materials	− 0.982	− 0.613	0.932	− 6.728	− 2.711	3.201
	(2.083)	(5.724)	(3.026)	(7.274)	(2.675)	(8.468)
**DID: Reading materials relative to none**	− 3.368	0.386	1.260	3.682	− 8.022	− 2.806
	(3.960)	(7.742)	(5.949)	(10.887)	(5.025)	(10.790)
TV/Radio/Mobile	− 3.932	− 2.449	− 7.121	− 4.566	− 1.791	− 0.589
	(2.678)	(4.639)	(4.529)	(7.004)	(3.137)	(6.407)
**DID: TV, Radio or Mobile at home relative to none**	− 2.291	− 1.611	− 3.471	6.991	− 0.868	− 7.409
	(3.985)	(6.339)	(6.613)	(9.446)	(4.835)	(8.773)
Controls for language	Yes	Yes	Yes	Yes	Yes	Yes
Other controls	Yes	Yes	Yes	Yes	Yes	Yes
Constant	17.155*	64.742**	21.983*	67.981**	11.580	69.721**
	(7.295)	(13.809)	(10.612)	(19.685)	(10.333)	(19.185)
Observations	665	665	359	359	306	306
R-squared	0.092	0.193	0.130	0.235	0.100	0.181

*Note:* Robust standard errors in parentheses (DID parameters) may be interpreted as the *relative loss* within factors. Controls for language and other controls included in the model (results not shown here). Asterisks *, ** indicate statistical significance at 5% and 1% level.

*Source:* CBE Monitoring and Evaluation 2016–2018.

***p < 0.01, **p < 0.05, *p < 0.1

For those who did report having learning activities or home support, we did not find any relative differences in literacy losses for children in the overall sample for letter identification (Table 3, Column 1). However, analysis by gender shows that boys who reported having access to learning activities at home had a smaller literacy loss in letter-sound identification during the transition period at home relative to boys who did not report having access to learning activities at home.

For reading comprehension, several significant factors emerged. First, boys and girls who asked adults for help with schoolwork at home had a smaller increase in zero scores for reading comprehension relative to those who did not ask for help. The relative difference is estimated at 26% for all children, 23% for boys, and 27% for girls. Second, children who reported having access to reading, writing, and counting activities at home also had a lower literacy loss in reading comprehension during the transition, with a 17.7% relative reduction in zero scores. We found that this result holds only for boys, whereby boys who had access to learning activities at home had a lower literacy loss in reading comprehension (22.5%) relative to other boys who did not have access to these activities at home.

## Combining factors: Literacy loss related to language, home learning support, and resources

Our final model brings together factors related to language preferences, home learning support, and resources. Since we must maintain a minimum cell count for estimation of these models with interactions, we only include here the interactions of the factors which were significant in prior estimates, as reported above. The first row of Table 4 shows the average literacy loss for children who did not prefer to learn in their mother tongue, did not find the language used by the teacher easy to understand, reported that the language used by the teacher was not the same to theirs, did not have support from adults with learning and did not have access to learning materials at home. For these children (without preferred language use in schools or home resource supports), the conditional average loss during the transition is estimated to be a 12.8% increase in zero scores for letter-sound identification (25.4% for boys and only 3.3% for girls) and 46.8% for reading comprehension (40.3% for boys and 50.5% for girls), as compared with those students with either preferred language use in schools or home resource supports. These are the largest estimates of any model thus far.

**Table 4:** Learning loss during transition time: relative zero scores in literacy using parsimonious model (by gender)

Variables	All		Boys		Girls	
	Letter identification	Reading comprehension	Letter identification	Reading comprehension	Letter identification	Reading comprehension
	[1]	[2]	[3]	[4]	[5]	[6]
Average learning loss (not preferred language; no home	12.775**	46.821**	25.364**	40.267**	3.298	50.547**
learning supports or resources)	(4.692)	(7.915)	(7.302)	(12.884)	(5.956)	(10.388)
Prefer to learn in MT	0.445	6.946*	1.359	1.076	− 0.045	10.64
	(1.949)	(4.131)	(2.690)	(6.242)	(2.892)	(5.664)
**DID: prefer MT relative to no MT**	2.130	− 15.715*	− 4.822	− 13.722	7.532	− 17.070*
	(3.566)	**(6.147)**	(5.243)	(9.360)	(4.815)	**(8.281)**
Language use by teacher easy to understand	0.190	3.190	1.902	2.441	− 0.811	4.800
	(2.172)	(4.308)	(3.563)	(6.207)	(2.520)	(6.003)
**DID: Language used by teacher easy relative to not**	− 0.283	− 11.757*	0.309	− 3.196	− 2.472	− 19.639*
	(3.330)	**(5.964)**	(5.229)	(9.035)	(4.230)	**(8.056)**
Language use by teacher same as child	2.735	11.652**	1.240	5.609	4.703	15.763**
	(2.288)	(4.335)	(3.397)	(6.245)	(3.069)	(6.047)
**DID: Same language used by teacher relative to not**	− 7.492*	− 9.413	− 7.330	− 4.721	− 6.663	− 13.339
	**(3.311)**	(6.017)	(4.781)	(9.047)	(4.582)	(8.183)
Ask for help most of the times	− 1.133	0.418	− 0.067	3.187	− 2.089	− 2.363
	(2.143)	(4.649)	(4.135)	(6.688)	(2.274)	(6.561)
**DID: most times ask relative to sometimes/never ask**	− 1.402	− 21.914**	0.219	− 22.865**	− 3.458	− 20.570*
	(3.461)	**(6.121)**	(5.473)	**(8.636)**	(4.485)	**(8.660)**
Literacy/numeracy activities	5.400*	14.198**	6.224	17.079*	5.728	12.706
	(2.260)	(4.904)	(3.626)	(6.652)	(2.967)	(7.050)
**DID: Learning activities relative to none**	− 7.563*	− 20.030**	− 16.424**	− 20.139*	− 0.319	− 18.398*
	**(3.488)**	**(5.629)**	**(5.497)**	**(8.250)**	(4.548)	**(7.872)**
Controls time to study, books at home, tv, radio, mobile	Yes	Yes	Yes	Yes	Yes	Yes
Other controls	Yes	Yes	Yes	Yes	Yes	Yes
Constant	17.135*	54.654**	19.02	58.407**	13.284	58.420**
	(7.355)	(13.819)	(10.813)	(19.696)	(10.389)	(19.187)
Observations	665	665	359	359	306	306
R-squared	0.096	0.209	0.136	0.239	0.105	0.210

*Note:* Robust standard errors in parentheses (DID parameters) may be interpreted as the *relative loss* within factors. Controls for time to study, books, tv, radio and mobile and other controls included in the model (results not shown here). Asterisks *, ** indicate statistical significance at 5% and 1% level.

*Source:* CBE Monitoring and Evaluation 2016–2018.

***p < 0.01, **p < 0.05, *p < 0.1

Overall, estimates from the combined model are similar to those obtained from separate models, with one interesting difference by gender. While there are slight changes in the magnitude of some literacy loss estimates, the implications remain virtually unchanged when the language factors are estimated with learning support and activities at home for boys. There were no relative differences in literacy loss during the transition according to language preference for boys. We found that boys who reported having support or learning activities at home achieved reductions in literacy loss for reading comprehension by 22.9% and 20.1%, respectively. In terms of letter identification, boys with learning activities at home showed a large reduction of 16.4%. For all these parameters, the size of the estimated relative literacy loss is substantial if one considers the scale of zero scores presented in Figure 1.

For girls, both language factors that were significant predictors of relative literacy loss for reading comprehension in the prior models remained significant in the combined model (with only slightly smaller magnitudes). Girls who preferred mother-tongue language instruction had a 17.1% reduction in zero scores relative to girls who did not prefer their mother tongue. Similarly, girls who found the language used by the teacher relatively easy to understand had a 19.6% reduction in zero scores relative to girls who did not find the language used by the teachers easier to understand. For home support, we continued to find that girls who were able to get support at home had significant reductions in zero scores relative to girls who did not have support at home (20.6%). While learning resources were not a significant predictor for girls in the previous model, we found that girls who had access to learning activities at home had a relatively smaller literacy loss in reading comprehension (18.4%) compared with girls who did not have access to these activities at home in our final model. Multiple comparison corrections (e.g. adjusted p-values) were not applied to these models. Since the focus of this study was to identify potential factors that may impact learning losses in order to inform future work, the decreased power and increased type II error rate (i.e., false negatives) that result from such corrections were not justifiable. Additionally, the magnitude of all significant coefficients in this study was large (pointing to their importance for discussion/consideration), and marginal statistical significance was not reported in any analysis.

## Discussion and conclusions

We are living in unprecedented times. Governments and school systems across the globe are faced with the task of providing educational opportunities to more than a billion children impacted by Covid-related school closures. Even as schools reopen, most continue to encounter new obstacles resulting from the need to incorporate social distancing and additional safety measures in systems that are designed for face-to-face teaching in typically crowded classrooms, hallways, and school grounds. As a result, many education systems are incorporating remote/distance learning to a larger degree, consequently requiring increased levels of support from parents and caregivers. However, there is little empirical evidence regarding the factors that may lead to differential effects on learning among students who will have to rely more heavily on parental support and teacher-free instruction than ever before. In this paper, we addressed this gap by examining the effects on learning of preferences for language of instruction and the availability of home learning support and home learning activities among Ghanaian students who participated in the CBE program and who spent 4 months out of school during the transition between the CBE program and the start of government school.

Overall, we found that large proportions of disadvantaged students who had attained foundational reading skills during the CBE program reverted to being nonperformers during their time away from school. Proportionally, we found that these losses were greater for basic skills, in our case, letter-sound identification, than they were for more advanced reading skills (i.e., reading comprehension). This result is consistent with previous studies on literacy loss during school holidays, which points to the larger skill loss for children who have not yet mastered foundational literacy skills (Education Endowment Foundation, 2020).

Reverting to being nonperformers during their time away from school was more pronounced for boys than for girls. Yet, when we introduced the role of preferences for language of instruction as reported by the students in our study, as well as the support they received at home with learning, the relative magnitude of literacy losses was higher for boys in letter identification but larger for girls in reading comprehension. For girls, significant reductions in foundational literacy loss were driven by those who preferred to learn in their own language, those who found the language used by the teacher easier to understand, those who consistently asked for help with work at home, and finally, those with access to learning activities at home. For boys, we did not find any of their views on language preference and usage by the teacher associated with reductions in literacy loss. However, we found significant reductions in loss driven by home support and access to learning activities at home. All of these factors reduced learning losses for reading comprehension, while fewer had an impact on reduced losses in letter identification.

We may infer some of the reasons for relative differences in literacy loss between boys and girls, particularly with respect to language preference. First, it is interesting to point out that we estimate a larger literacy loss for boys during the transition period but note that they bounce back better than girls after this transition period. This result is consistent with Carter et al. (2020a), who demonstrated that low-achieving girls are at particular risk of remaining low achievers, whereas low-achieving boys are more likely to catch up. There are differences between boys and girls in their engagement with work activities outside of the home (with boys being more likely to work outside of the house)—which may explain their higher literacy loss during time away from school (Akyeampong et al., 2018).

Recent studies have suggested effective ways to stem the academic learning loss using a variety of resources, including digital technologies and radio (Alasuutari, 2020; Azevedo et al., 2020). However, there is also recognition that many of these are likely to increase inequality in learning continuity because of inequitable access to these resources (UNICEF, 2020). This is further supported by our own findings on differential access to home supports and the inequities in reading outcomes that they impact. Resources in the form of print material to both children and households may offer a more equitable opportunity to ensure learning continuity even for the poorest households with limited literacy (Mundy and Hares, 2020).

Our results show foundational literacy learning is being eroded during the four-month school holiday period in Ghana, which confirms what other recent studies suggest in terms of foundational learning losses during school closures. Foundational learning loss due to the time out of school is likely to be particularly significant for children from poor and disadvantaged backgrounds (Wagner et al., 2018). For these children, low academic achievement after the transition could increase their risk of dropping out of school (Selbervik, 2020). In addition, there can be cumulative future effects from school closures, including lower chances of continuing in education to upper secondary and tertiary levels, reduced earnings and labor market potential, as well as future impacts of health and wellbeing (Mundy and Hares, 2020). In effect, long school closures pose a serious risk to reducing inter-generational poverty.

Our results also suggest that in tackling foundational learning loss, a one-size-fits-all approach may not actually meet the needs of everyone. There is always a diversity of learning experiences prior to school closure and in the transition period. Some students will suffer more from a lack of home support for learning, which is then compounded if they struggled to understand their lessons due to the language of instruction used in their school.

In qualitative analyses of learning experiences of the CBE children after transition, Akyeampong et al. (2018) found that those who had been taught using their own language showed stronger continuity in learning after transition. They were also more confident and optimistic in their ability to make progress in learning. Notably, low-performing boys and girls showed greater “anxiety and frustration at their inability to understand or participate and expressed fear of humiliation if this was publicly revealed” (Akyeampong et al., 2018, p. 2). Those children who developed the least foundational literacy skills and have been taught in an unfamiliar language are at a greater risk of slower recovery after transition.

As our results have demonstrated, widening foundational literacy gaps could be expected for students who do not receive teaching and support in a language that they understand or who do not have the resources, support, or activities at home to continue to learn. While boys have larger losses in literacy, other research has shown that they are more likely to bounce back more rapidly (Carter et al., 2020a). Therefore, there is an even greater concern for girls who are likely to fall behind and potentially make the slowest recovery. Both results suggest that schools and teachers must pay closer attention to recovering children’s learning losses, ensure that language of instruction is not a barrier to this recovery, and consider the interplay of gender, language, and household dynamics in the learning recovery of all children. With recurring school closures and a new reliance on alternative learning opportunities for children, these factors are increasingly essential to reduce inequities and support continued learning for all children.
